# On-Demand Free Radical Release by Laser Irradiation for Photothermal-Thermodynamic Biofilm Inactivation and Tooth Whitening

**DOI:** 10.3390/gels9070554

**Published:** 2023-07-07

**Authors:** Qi Zhang, Yuan Liu, Meng Ding, Lihui Yuwen, Lianhui Wang

**Affiliations:** 1 State Key Laboratory of Organic Electronics and Information Displays, Jiangsu Key Laboratory for Biosensors, Institute of Advanced Materials (IAM), Nanjing University of Posts and Telecommunications, Nanjing 210023, China; zhangqi_nj@163.com (Q.Z.); lyy657200@163.com (Y.L.); 2 Nanjing Stomatological Hospital, Medicine School, Nanjing University, Nanjing 210008, China; xzdingmeng@163.com

**Keywords:** photothermal-thermodynamic therapy, controllable free radical release, bacterial biofilm, bacterial biofilm, tooth whitening, transition-metal dichalcogenides

## Abstract

Dental diseases associated with biofilm infections and tooth staining affect billions of people worldwide. In this study, we combine photothermal agents (MoS_2_@BSA nanosheets, MB NSs), a thermolysis free-radical initiator (AIPH), and carbomer gel to develop laser-responsive hydrogel (MBA-CB Gel) for biofilm inactivating and tooth whitening. Under a physiological temperature without laser irradiation, MB NSs can eliminate free radicals generated from the slow decomposition of AIPH due to their antioxidative activity, thereby avoiding potential side effects. A cytotoxicity study indicates that MB NSs can protect mammalian cells from the free radicals released from AIPH without laser irradiation. Upon exposure to laser irradiation, MB NSs promote the rapid decomposition of AIPH to release free radicals by photothermal effect, suggesting their on-demand release ability of free radicals. In vitro experimental results show that the bacteria inactivation efficiency is 99.91% (3.01 log units) for planktonic *Streptococcus mutans* (*S. mutans*) and 99.98% (3.83 log units) for planktonic methicillin-resistant *Staphylococcus aureus* (MRSA) by the mixed solution of MB NSs and AIPH (MBA solution) under 808 nm laser irradiation (1.0 W/cm^2^, 5 min). For *S. mutans* biofilms, an MBA solution can inactivate 99.97% (3.63 log units) of the bacteria under similar laser irradiation conditions. Moreover, MBA-CB Gel can whiten an indigo carmine-stained tooth under laser irradiation after 60 min of laser treatment, and the color difference (Δ*E*) in the teeth of the MBA-CB Gel treatment group was 10.9 times that of the control group. This study demonstrates the potential of MBA-CB Gel as a promising platform for biofilm inactivation and tooth whitening. It is worth noting that, since this study only used stained models of extracted teeth, the research results may not fully reflect the actual clinic situation. Future clinical research needs to further validate these findings.

## 1. Introduction

Dental diseases affect more than 2.3 billion people worldwide, resulting in long-lasting physical and psychological complications for patients, particularly for those living in impoverished and medically underserved areas [1,2,3]. Specifically, tooth discoloration and dental biofilm infections are the primary causative factors of dental diseases [4]. On one hand, poor oral hygiene and dietary habits such as smoking, drinking certain beverages (e.g., coffee and tea), or consuming specific foods can lead to discoloration and staining of teeth [5,6,7]. On the other hand, a contaminated tooth facilitates bacterial attachment, eventually becoming a breeding ground for bacterial colonization and leading to changes in the oral microbial community [8]. These attached bacteria secrete extracellular polymeric substances (EPS) to form biofilms, which further exacerbate more severe dental diseases such as dental plaque, enamel demineralization, and periodontitis [9,10,11]. Clinically, high concentrations of carbamide peroxide and hydrogen peroxide are commonly employed for tooth bleaching [12,13,14]. Although these bleaching agents provide efficient whitening effects, the use of high-concentration peroxides often leads to serious side effects, such as tooth mineral loss and dental hypersensitivity, as well as an increased risk of biofilm recolonization [15,16]. Consequently, developing an effective and non-invasive tooth whitening strategy that can both degrade colored substances and inactivate dental bacterial biofilms is essential.

Free radicals are molecular fragments or molecules with an unpaired valence electron, demonstrating high reactivity [17,18,19]. Under normal conditions, free radicals play an essential role in maintaining cellular metabolism homeostasis [20]. However, excessive accumulation of free radicals can react with organic molecules, such as DNA, proteins, and lipids, resulting in cellular dysfunction [21]. Therefore, highly reactive free radicals have been extensively applied to cancer and bacterial infection treatments [22,23,24,25]. For example, photodynamic therapy (PDT) serves as an effective adjunctive approach in clinical dental treatments, which can prevent primary and secondary injuries to hard and soft tissues [26]. The 2, 2′-azobis[2-(2-imidazolin-2-yl) propane] dihydrochloride (AIPH) is a thermally decomposable radical initiator that is stable at low temperatures but can decompose under thermal activation, offering an alternative thermodynamic strategy for pathogenic bacteria inactivation and stained molecules degradation [27,28]. However, the limited stability of AIPH under physiological conditions raises concerns about the uncontrolled production of free radicals and potential safety hazards. Thus, the development of a controllable free radical release strategy is crucial for achieving on-demand release. To address this issue, we propose the use of MoS_2_ nanosheets (MoS_2_ NSs) to control the free radical release. With their exceptional near-infrared (NIR) photothermal properties, MoS_2_ NSs can be heated from laser irradiation to rapidly initiate the generation of sufficient free radicals from AIPH [29,30,31]. Moreover, MoS_2_ NSs exhibit a remarkable ability to reduce excess alkyl radicals without stimulation from NIR laser irradiation, thereby avoiding safety risks associated with the spontaneous decomposition of AIPH [32,33].

**Scheme 1:**
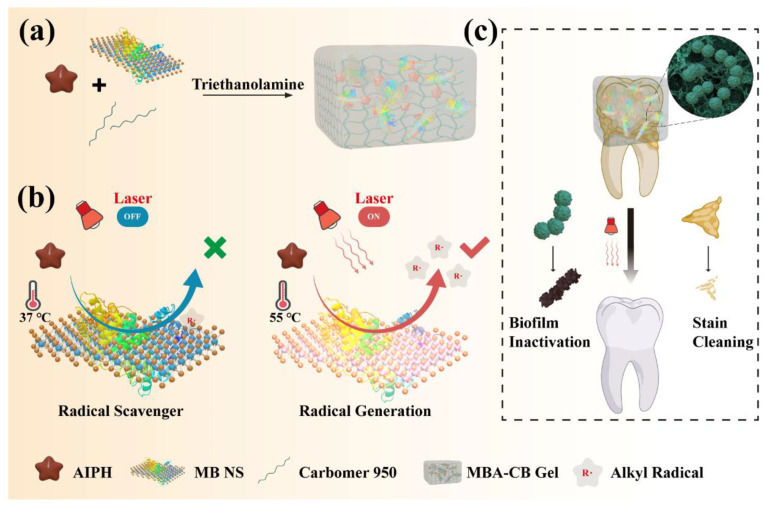
(**a**) Preparation of carbomer gels containing MB NSs and AIPH (MBA-CB Gel). (**b**) On-demand release of alkyl free radicals from AIPH by MB NSs with laser on/off. (**c**) Applications of MBA-CB Gel for biofilm inactivation and tooth whitening.

In this study, we harnessed the distinctive properties of MoS_2_ NSs to develop a laser-responsive hydrogel for photothermal-thermodynamic treatment of biofilm and tooth staining (Scheme 1). To begin with, MoS_2_ NSs were synthesized by using an ultrasound-assisted lithium intercalation method and subsequently modified with bovine serum albumin (BSA) to create MoS_2_@BSA nanosheets (MB NSs) [34,35]. At physiological temperature (37 °C), MB NSs employ their antioxidative properties to eliminate free radicals produced by AIPH, consequently reducing the risk of side effects. Upon laser exposure, the photothermally responsive MB NSs experience a rapid temperature increase, which in turn facilitates AIPH decomposition and free radical release. By utilizing MB NSs and AIPH with 808 nm laser irradiation, *S. mutans* both in planktonic and biofilm forms can be effectively inactivated. Furthermore, by applying a carbomer composite hydrogel containing MB NSs and AIPH (MBA-CB Gel) to the tooth surface and subjecting it to laser irradiation, the indigo carmine-stained tooth can be rapidly whitened. In conclusion, this combined therapy offers a highly efficient and controllable method for addressing tooth-associated biofilms and discoloration issues.

## 2. Results and Discussion

### 2.1. Preparation of MB NSs

**Figure 1:**
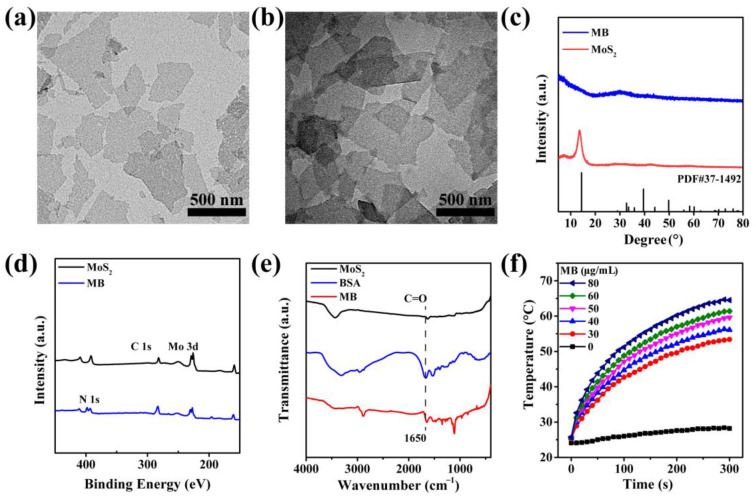
Characterization of MB NSs. Transmission electron microscopy (TEM) images of MoS_2_ NSs (**a**), and MB NSs (**b**). (**c**) X-ray photoelectron spectroscopy (XPS) spectra of MoS_2_ NSs and MB NSs. (**d**) X-ray diffraction (XRD) patterns of MoS_2_ NSs and MB NSs. (**e**) Fourier transform infrared spectroscopy (FT-IR) spectra of MoS_2_ NSs, BSA, and MB NSs. (**f**) Photothermal heating curves of MB NSs aqueous dispersions at various concentrations under 808 nm laser irradiation (1.0 W/cm^2^).

In this study, we synthesized MoS_2_ NSs using an ultrasonication-enhanced lithium intercalation method, following the procedure outlined in our previous work [35]. As shown in Figure 1a, the as-prepared MoS_2_ NSs showed uniform sheet-like morphology. After being modified with BSA, the MB NSs retained similar morphology to the unmodified MoS_2_ NSs, with no evident breakage observed (Figure 1b). The X-ray diffraction (XRD) pattern presents the diffraction lines near 14°, which can be assigned to the (002) plane for MoS_2_ NSs (Figure 1c) [36]. Due to BSA modification causing an increase in the distance between MoS_2_ layers, the diffraction peak near 14° was no longer observed in the XRD pattern of the MB NSs. Besides, the wide diffraction peak near 28° was observed in the XRD pattern of MB NSs [37]. As shown in the X-ray photoelectron spectroscopy (XPS) spectra of MB NSs presented in Figure 1d, there was a decrease in the characteristic binding energy peak of Mo 3d (232 eV). In contrast, the characteristic binding energy peak of N 1s (399 eV) increased after the surface of MoS_2_ NSs was coated with BSA. The Fourier-transform infrared spectroscopy (FT-IR) spectra of MB NSs displayed IR absorption bands at 1650 cm^−1^, attributed to the existence of the C=O group from BSA (Figure 1e) [34,37], which confirms that BSA was successfully coated onto the surface of MoS_2_ NSs. The photothermal effect of MB NSs aqueous dispersions was further evaluated. As shown in Figure 1f, the temperature of MB NSs aqueous dispersions increased gradually under 808 nm laser irradiation. In contrast, the temperature of water showed a limited increment under the same condition. When the concentration of MB NSs was 40 μg/mL, the temperature of dispersions increased to 55 °C after 5 min of exposure to the 808 nm laser, indicating the excellent photothermal effect of MB NSs.

### 2.2. Evaluating the Stability of AIPH and Its Dye Degradation Capability

**Figure 2:**
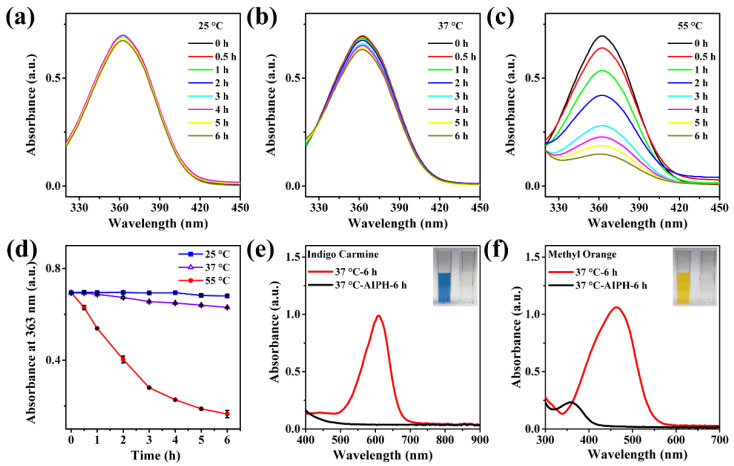
The stability of AIPH at various temperatures and the degradation effect of various dyes by AIPH. (**a**–**c**) Ultraviolet-visible (UV-Vis) absorption spectra of AIPH aqueous solutions incubated at 25 °C (**a**), 37 °C (**b**), and 55 °C (**c**) for different times. (**d**) The absorbance of AIPH aqueous solutions at 363 nm at different temperatures for different times (*n* = 3). (**e**,**f**) Ultraviolet-visible near-infrared (UV-Vis-NIR) absorption spectra and photographs (inset) of indigo carmine (**e**) and methyl orange (**f**) under various incubation conditions.

The stability of AIPH is temperature-dependent. As shown in Figure 2a,d, AIPH exhibited a certain degree of stability under relatively low-temperature conditions (25 °C). After 6 h of incubation at 37 °C, approximately 9.3% of AIPH decomposed (Figure 2b,d). As the temperature increased to 55 °C, AIPH underwent rapid decomposition, with nearly 76.2% decomposed after incubation for 6 h (Figure 2c,d). Importantly, AIPH already displayed significant decomposition at physiological temperature (37 °C) and generated free radicals. As shown in Figure 2e,f and Figure S2, various dyes, including indigo carmine, methyl orange, methylene blue, and rhodamine B were significantly degraded at 37 °C after 6 h incubation [38,39,40,41]. These results indicate that AIPH is unstable under physiological temperature conditions and may result in unpredictable side effects.

### 2.3. Regulating the Release of Alkyl Free Radicals with MB NSs

**Figure 3:**
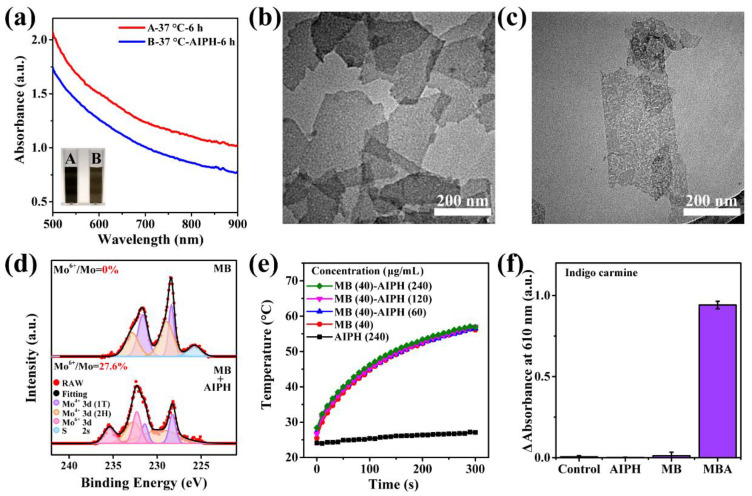
The regulatory capability of MB NSs on free radical release. (**a**) The UV-Vis-NIR absorption spectra of MB NSs aqueous solutions after incubation with AIPH at 37 °C (inset: photographs of MB NSs and MB NSs + AIPH). TEM images of MB NSs after being incubated for 6 h at 37 °C without (**b**) and with AIPH (**c**). (**d**) XPS spectra of MB NSs after 6 h incubation at 37 °C with and without AIPH. (**e**) Temperature evolution curves of MB NSs aqueous dispersions with varying AIPH concentrations. (**f**) NIR light-triggered degradation of indigo carmine (50 μg/mL) by MB NSs mixed with AIPH (MB NSs 40 μg/mL, AIPH 60 μg/mL). Irradiation conditions were kept constant (808 nm, 1.0 W/cm^2^, 5 min) across all experimental groups.

As shown in Figure 3a, when MB NSs aqueous dispersions were incubated with AIPH at 37 °C for 6 h, a significant color fading and a decrease in absorbance between 500–900 nm were observed, in contrast to the minimal change in the pure MB NSs aqueous dispersions alone. In Figure 3b,c, TEM images showed a significant increase in the number of surface cracks on the MB NSs after co-incubation with AIPH. Referring to Figure 3d, XPS analysis reveals that following incubation with AIPH, the percentage of high-valence Mo element (Mo^6+^, binding energy 225 eV) within MB NSs experienced an increase from nearly 0% to 27.6% [42]. Subsequently, we employed 2, 2′-azino-bis(3-ethylbenzothiazoline-6-sulfonic acid) (ABTS) as an indicator to further assess the capability of MB NSs for scavenging free radicals [43,44]. As shown in Figure S3, the production of alkyl free radicals was significantly reduced in the presence of MB NSs. Additionally, when MB NSs (40 μg/mL) were added, AIPH aqueous solutions displayed minimal cytotoxicity towards L-O2 cells, even at concentrations reaching 240 μg/mL (Figure S5), indicating the antioxidative property of MB NSs and its capacity to eliminate excess free radicals [42]. To assess the potential of MB NSs in promoting free radical release under laser irradiation, we initially tested the photothermal performance of MB NSs aqueous dispersions. Figure 3e shows that MB NSs have remarkable photothermal performance with negligible influence of AIPH. When exposed to 808 nm laser irradiation (1.0 W/cm^2^), the mixture of MB NSs and AIPH (MB NSs 40 μg/mL, AIPH 60 μg/mL) reached 55 °C within 5 min, while the AIPH solution showed no significant heating under identical conditions. Next, to further confirm the photothermally activated radical generation, we employed indigo carmine (50 μg/mL) as an indicator. As illustrated in Figure 3f and Figure S4, the combination of MB NSs and AIPH effectively degraded the dye within 5 min. Under the same condition, neither pure AIPH (60 μg/mL) nor MB NSs (40 μg/mL) can independently degrade the dye. These research findings suggest that MB NSs can facilitate AIPH decomposition and free radical release under laser irradiation, offering the potential for controlled radical modulation in light-responsive applications.

### 2.4. Treatment of Planktonic MRSA and S. mutans In Vitro

**Figure 4:**
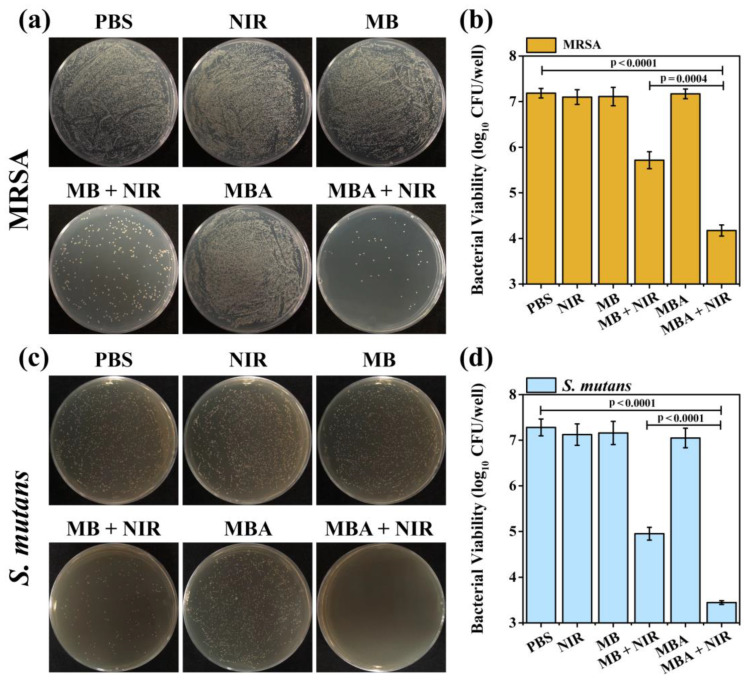
Treatment of planktonic MRSA and *S. mutans*. (**a**) Photographs of MRSA colonies formed on Luria–Bertani (LB) agar plates and the corresponding numbers of MRSA (**b**) after various treatments. (**c**) Photographs of *S. mutans* colonies formed on Brain Heart Infusion Broth (BHI) agar plates and the corresponding numbers of *S. mutans* (**d**) after various treatments (*n* = 3 biologically independent samples; mean ± SD). All experimental groups were subjected to identical irradiation conditions (808 nm, 1.0 W/cm^2^, 5 min). Statistical significance was analyzed via one-way ANOVA with a Tukey post hoc test.

The antibacterial effect of the solution containing MB NSs and AIPH (short for MBA) was evaluated using both planktonic (MRSA) and planktonic *S. mutans*. As illustrated in Figure 4a,b, the bacterial inactivation efficiency of the MB NSs under laser irradiation is approximately 96.59% (1.46 log units) against MRSA, which suggests that MB NSs can inactivate bacteria via a photothermal effect. In comparison, the MBA solution without NIR laser irradiation exhibited a negligible antibacterial effect, which can be ascribed to the fact that most free radicals generated by AIPH were scavenged by MoS_2_. After laser irradiation, the MBA solution killed 99.91% (3.01 log units) of MRSA, which is significantly higher than MB NSs and MBA without laser irradiation, suggesting the photothermal effect can activate the decomposition of AIPH for further thermodynamic therapy. As shown in Figure 4c,d, the MBA solution under laser irradiation can kill 99.98% (3.83 log units) of planktonic *S. mutans*, which is much higher than the MB NSs + NIR (99.53%, 2.33 log units). MBA solution without exposure to laser irradiation exhibits no noticeable antibacterial effects. The above results further confirm the excellent antibacterial effect of the combination of the photothermal effect of MoS_2_ and the thermodynamic effect of AIPH.

### 2.5. Treatment of S. mutans Biofilm In Vitro

**Figure 5:**
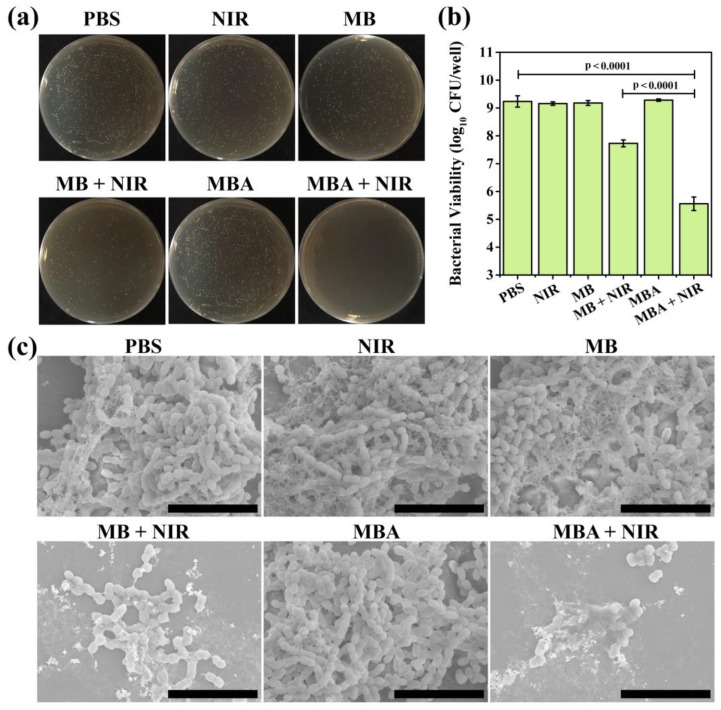
Treatment of *S. mutans* biofilms. (**a**) Photographs of *S. mutans* colonies formed on BHI agar plates and the corresponding numbers of *S. mutans* (**b**) from the biofilm after various treatments (*n* = 3 biologically independent samples, mean ± SD). (**c**) SEM images of *S. mutans* after various treatments. All experimental groups were subjected to an identical irradiation condition (808 nm, 1.0 W/cm^2^, 5 min). The scale bar is 3 μm. Statistical significance was analyzed via one-way ANOVA with a Tukey post hoc test.

The anti-biofilm effect of MBA was further assessed using an in vitro *S. mutans* biofilm model. As shown in Figure 5a,b, after laser irradiation, MB NSs exhibited limited anti-biofilm activity against the *S. mutans* biofilm, inactivating approximately 96.59% (1.46 log units) of *S. mutans* within the biofilm. However, upon incorporating AIPH, the combined interaction between MB NSs and AIPH led to a significant improvement in antibacterial performance, inactivating nearly 99.97% (3.63 log units) of *S. mutans* within the biofilm after laser irradiation. Moreover, scanning electron microscopy (SEM) images indicate that MBA with laser irradiation can significantly destroy the bacteria within the biofilm (Figure 5c). In further characterization, confocal laser scanning microscopy (CLSM) images demonstrated that the photothermal and thermodynamic therapy based on MBA can effectively inactivate bacteria within the *S. mutans* biofilm (Figure S6). The results of numerous antibacterial tests have demonstrated that the synergistic treatment strategy combining photothermal therapy and thermodynamic therapy is more efficient and offers broader application prospects compared to single-modal treatment methods. Additionally, compared to traditional surgical and antibiotic treatments, NIR phototherapy boasts advantages such as no drug resistance issues, high efficiency, minimal invasiveness, and high controllability [5,38,45].

### 2.6. Preparation of MB NSs-AIPH-Carbomer Composite Hydrogel (MBA-CB Gel)

**Figure 6:**
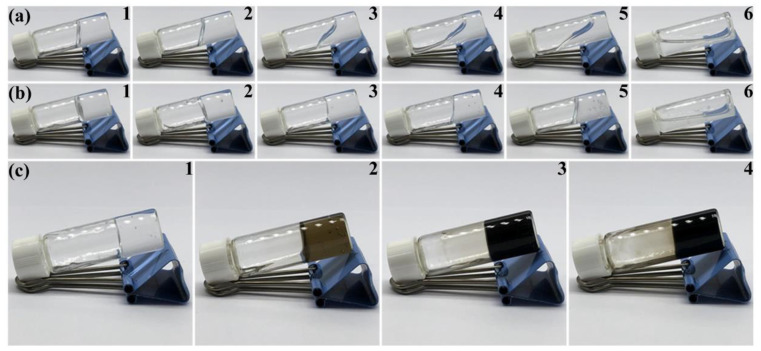
Images of carbomer gels with different components. (**a**) The gel formation under different AIPH feeding concentrations (samples (**1**–**6**): 0.12, 0.24, 0.48, 0.96, 2.5, and 5.0 mg/mL) when the final concentration of carbomer was 0.5%. (**b**) The gel formation under different AIPH feeding concentrations (samples (**1**–**6**): 0.12, 0.24, 0.48, 0.96, 2.5, and 5.0 mg/mL) when the final concentration of carbomer is 1.0%. (**c**) The gel formation under different MB NSs feeding concentrations (samples (**1**–**4**): 0, 50, 100, and 150 μg/mL) when the carbomer concentration was 1.0% and AIPH concentration was 2.5 mg/mL.

To study the tooth whitening effect, MB NSs and AIPH were combined with carbomer (CB) to form MB NSs-AIPH-carbomer composite hydrogel (MBA-CB Gel). First, the preparation conditions on the gelation were tested. As shown in Figure 6, the concentrations of carbomer and AIPH had a significant influence on the gel formation, whereas the inclusion of MB NSs exerted no notable effect on the gelation process. As shown in Figure 6(a1–a6), when the concentration of carbomer was fixed at 0.5%, the flowability of the composite increased noticeably with increasing AIPH concentration (0, 60, 120, 240, 480, 960 μg/mL). At an AIPH concentration of 240 μg/mL (Figure 6(a4)), the composite slid down the tube walls, barely forming a gel. Meanwhile, in Figure 6(b1–b6), when the concentration of carbomer was fixed at 1.0%, the flowability of the composite hydrogel slightly increased with increasing AIPH concentration (0, 0.24, 0.48, 0.96, 2.5, 5.0 mg/mL). When the AIPH concentration was 2.5 mg/mL (Figure 6(b5)), the composite still maintained a gel state. As illustrated in Figure 6(c1–c4), with the increasing MB NSs loading (0, 50, 100, 150 μg/mL), the color of the MBA-CB hydrogel gradually deepened, while maintaining a gel form throughout.

**Figure 7:**
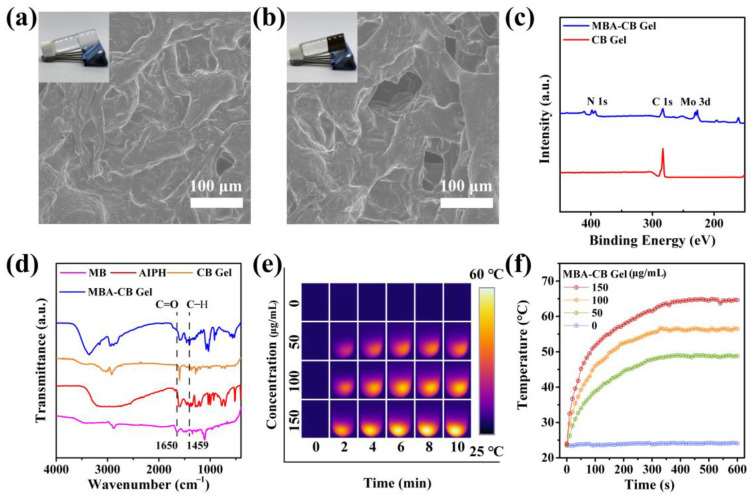
Characterization of MBA-CB Gel. (**a**) SEM images and pictures (inset) of carbomer gel (CB Gel) and (**b**) carbomer composite gel encapsulating MB NSs and AIPH (MBA-CB Gel). (**c**) XPS spectra of CB Gel and MBA-CB Gel. (**d**) FT-IR spectra of MB NSs, AIPH, CB Gel, and MBA-CB Gel. (**e**) Infrared thermal images and (**f**) corresponding temperature increase curves of MBA-CB Gel with different MB NSs contents (0, 50, 100, 150 μg/mL). Irradiation conditions were kept constant (808 nm, 1.0 W/cm^2^, 5 min) across all experimental groups.

We further characterized the morphology and properties of the composite hydrogel. As the SEM images show (Figure 7a,b), the carbomer hydrogel encapsulated MB NSs and AIPH, forming a uniform texture. The XPS analysis presented in Figure 7c reveals that MBA-CB Gel showed the emergence of Mo 3d peak (binding energy 232 eV) from MB NSs and N 1s peak (binding energy 399 eV) originating from AIPH and BSA, while these peaks were absent in the CB Gel. This finding confirms the loading of both MB NSs and AIPH into the MBA-CB Gel. In Figure 7d, when compared to the CB Gel, the FT-IR spectrum of the MBA-CB Gel exhibits an additional IR band at 1650 cm^−1^, originating from BSA (C=O bending vibration), which indicates the presence of MB NSs within the hydrogel. Furthermore, the emergence of the IR band at 1459 cm^−1^ (C–H stretching vibration absorption) provided additional evidence for AIPH’s presence [31]. These results provide strong support for the successful preparation of the MBA-CB Gel. To explore the potential application of the composite hydrogel in laser-induced dental whitening applications, we first examined the photothermal performance of the MBA-CB Gel. As demonstrated in Figure 7e,f, MBA-CB Gel displayed excellent NIR photothermal properties. When the MB NSs loading reached 100 μg/mL and was subjected to laser irradiation (808 nm, 1.0 W/cm^2^), the MBA-CB Gel rapidly heated to 55 °C within 2 min and maintained this temperature for 10 min. Under the same conditions, the temperature change for the CB Gel was negligible.

### 2.7. Tooth Whitening via Laser-Induced Photothermal-Thermodynamic Treatment

**Figure 8:**
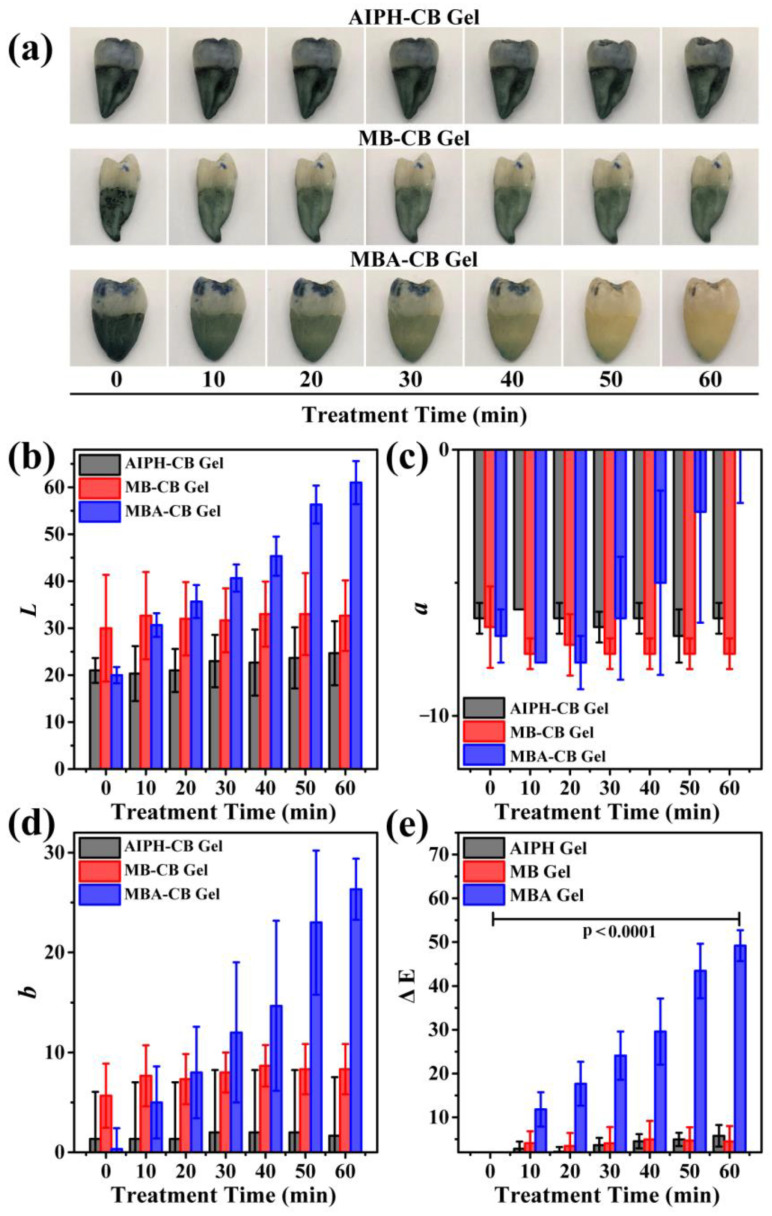
Tooth whitening by AIPH-CB Gel, MB-CB Gel, and MBA-CB Gel. (**a**) Photographs of indigo carmine-stained teeth after treatment with various CB gels under laser irradiation at different time points. Tooth whitening performance was evaluated using variations in luminance *L* (**b**), color value of the red-green axis *a* (**c**), color value of the blue-yellow axis *b* (**d**), and color difference Δ*E* (**e**) calculated from (**a**). Statistical significance was analyzed via one-way ANOVA with a Tukey post hoc test.

The potential application of MBA-CB Gel in tooth whitening was further assessed. Initially, teeth were stained by immersing them in a 10 mg/mL indigo carmine solution for one week. Subsequently, the discolored teeth were individually set into test tubes, each containing 2 mL of carbomer hydrogel with AIPH (AIPH-CB Gel), carbomer hydrogel with MB NSs (MB-CB Gel), or carbomer hydrogel with MBA (MBA-CB Gel), respectively. After placement, the teeth were irradiated by an 808 nm laser at 1.0 W/cm^2^. Tooth images were captured every 10 min to record color changes, with a treatment duration of 1 h. As demonstrated in Figure S7, the temperature increase for teeth in both the MB-CB Gel and MBA-CB Gel treatment groups was substantially significant, reaching 56 °C and 58 °C, respectively, whereas teeth treated with AIPH-CB Gel only experienced an increase to 33 °C. As indicated in Figure 8a, after treatment, teeth treated with AIPH-CB Gel exhibited almost no change in surface color. Teeth treated with MB-CB Gel experienced a slight lightening of their surface color. Meanwhile, teeth treated with MBA-CB Gel displayed a significant lightening of their surface color upon laser irradiation. To further quantify the tooth-whitening effect of MBA-CB Gel, we employed the International Commission on Illumination (CIELab) system to analyze the color alterations of stained teeth [5,46,47]. Figure 8b–e demonstrates that the increase in *L* value for teeth treated with MBA-CB Gel was the most prominent, while the increases of *a* and *b* were also the most significant. The color difference (Δ*E*) for the MBA-CB Gel treatment group was 8.5 times and 10.9 times greater compared to the AIPH-CB Gel and MB-CB Gel treatment groups, respectively, which highlights the excellent tooth whitening performance of MBA-CB Gel upon laser irradiation. In future studies, bioinspired hydroxyapatite in toothpaste form (zinc-hydroxyapatite) may be introduced to improve the deposition of calcium phosphate and enhance antibacterial action [48,49].

## 3. Conclusions

In summary, we develop a laser-responsive photothermal-thermodynamic treatment strategy by fully utilizing the photothermal properties and antioxidant capabilities of MB NSs to achieve the on-demand release of alkyl free radicals from AIPH. This approach not only efficiently inactivated *S. mutans* biofilms, but also achieved efficient tooth whitening while avoiding the potential safety risks associated with excess free radicals. Under physiological temperatures, MB NSs could eliminate free radicals generated by the spontaneous decomposition of AIPH, ensuring biocompatibility. Upon laser irradiation, MB NSs induce a temperature increase, promoting AIPH decomposition and free radical release, achieving efficient bacterial inactivation and dye degradation. In vitro antibacterial experiments demonstrated that MB NSs combined with AIPH, irradiated by a near-infrared laser, not only effectively inactivated 99.98% (3.83 log units) of planktonic *S. mutans*, but also killed 99.97% (3.63 log units) of the bacteria within the *S. mutans* biofilm. Furthermore, MB NSs and AIPH were integrated into carbomers to form the MBA-CB Gel that could adhere to tooth surfaces. Upon NIR-laser irradiation, the tooth surface pigmentation was significantly degraded by MBA-CB Gel, achieving highly efficient tooth whitening. Consequently, this study demonstrates an intelligent radical release hydrogel that can effectively eradicate bacterial biofilms and whiten colored teeth by a photothermal-thermodynamic treatment strategy, which demonstrates the substantial potential for treating dental-related diseases. In future clinical practices, combining photothermal and thermodynamic treatments with preventive hydroxyapatite mouthwash and toothpaste is expected to further decrease the risk of dental caries occurrence and progression [50].

## 4. Materials and Methods

### 4.1. Materials

Molybdenum (IV) sulfide (MoS_2_) powder (<2 μm, 99%), 2, 2′-azobis[2-(2-imidazolin-2-yl) propane] dihydrochloride (AIPH), 2, 2′-azino-bis(3-ethylbenzothiazoline-6-sulfonic acid) (ABTS), carbomer 940, and bovine serum albumin (BSA) were obtained from Sigma-Aldrich. The n-butyllithium (n-BuLi, 2.4 M hexane solution) was charged from Amethyst. The triethanolamine, indigo carmine, methyl orange, methylene blue, and rhodamine B were brought from Sinopharm Chemical Reagent Co., Ltd. (Shanghai, China). Ultrapure water (Millipore, Burlington, MA, USA, 18.2 MΩ) was used to prepare the aqueous solutions.

### 4.2. Characterization

The morphology of MoS_2_ NSs, MB NSs, and MBA-CB Gel was characterized by a transmission electron microscope (TEM, HT7700, Hitachi, Tokyo, Japan) and a scanning electron microscope (SEM, Hitachi S-4800, Tokyo, Japan). X-ray diffraction (XRD) patterns were recorded on a diffractometer (D8 Advance A25, Bruker, Karlsruhe, Germany) with Cu Kα radiation (λ = 1.5418 Å). X-ray photoelectron spectroscopy (XPS) was recorded on a PHI 5000 Versa Probe (Ulvac-Phi, Chigasaki, Japan) with Al Kα (hν = 1486.6 eV) to study the compositions of the materials. Ultraviolet-visible near-infrared (UV-Vis-NIR) absorption spectrophotometer (UV-3600, Shimadzu, Kyoto, Japan) and a microplate spectrophotometer (PowerWave XS2, BioTek, Winooski, VT, USA) were used. The photothermal effect was measured using an 808 nm laser (MIL-N-808, CNI Changchun, China), with the power density of laser irradiation measured by a digital power meter (PM100D, Thorlabs, Newton, NJ, USA). The temperature change and IR thermal photos were recorded using an infrared thermal camera (Fortic225, FOTRIC, Shanghai, China).

### 4.3. Preparation of MB NSs

The preparation of MoS_2_ NSs used the ultrasonication-enhanced lithium intercalation method according to our previous work [35]. The as-prepared MoS_2_ NSs (10 mg) were added with 200 mg BSA in 25 mL water and stirred at 1200 rpm under room temperature for 12 h. The mixture was centrifuged at 12,000 rpm three times to remove the unreacted BSA, and finally obtain MB NSs.

### 4.4. Preparation of MBA-CB Gel

Mix the carbomer aqueous solution (1%, 1 mL) with AIPH aqueous solution (10 mg/mL, 500 μL) and MB NSs aqueous dispersion (400 μg/mL, 500 μL), and vigorously vortex for 60 s. Subsequently, add a drop of triethanolamine to the mixture; and vortex vigorously for 90 s. Finally, the mixture coagulates and forms the black MBA-CB Gel.

### 4.5. Evaluating the Stability of AIPH at Various Temperatures and the Degradation Effect of Released Alkyl Radicals on Dyes

First, the absorbance at 363 nm of AIPH aqueous solutions with various concentrations was measured, and the standard calibration curve was plotted.. Next, 5 mL of 2.5 mg/mL AIPH solution was incubated at 25 °C, 37 °C, and 55 °C with agitation. Throughout the process from 0 to 6 h, the absorbance values at 363 nm of AIPH aqueous solutions at different temperatures were recorded. Finally, evaluate the efficiency of AIPH in degrading dyes at 37 °C using four types of dyes: indigo carmine (50 μg/mL, absorption peak at 610 nm), methylene blue (5 μg/mL, absorption peak at 663 nm), methyl orange (15 μg/mL, absorption peak at 460 nm), and rhodamine B (10 μg/mL, absorption peak at 554 nm). To reduce the impact of errors and improve the reliability of results, three independent replicates were generally made for in vitro experiments.

### 4.6. MB NSs Scavenging Alkyl Radicals at Physiological Temperature

To assess the capacity of MB NSs (50 μg/mL) to neutralize free radicals released from AIPH (2.5 mg/mL) at 37 °C, the two solutions were combined and incubated for 6 h at 37 °C. Following incubation, absorption spectra, morphological changes, and composition alterations of MB NSs were examined. The reaction between ABTS and AIPH-generated free radicals leads to the formation of ABTS^+^•, displaying distinct absorption between 400 to 1000 nm. As a result, ABTS serves as a chemical probe to further validate the radical scavenging efficacy of MB NSs. In the experiment, water, MB NSs aqueous dispersion (20 μg/mL), AIPH aqueous solution (2.5 mg/mL), and a mixture of AIPH and MB NSs aqueous solution were combined with ABTS (1 mg/mL) aqueous solution and incubated at 25 °C and 55 °C through oscillation for 20 min. After incubation, the UV-Vis-NIR absorption spectrum of each sample was measured using a spectrophotometer. To reduce the impact of errors and improve the reliability of the results, three independent replicates were generally made for in vitro experiments.

### 4.7. MB NSs Promote AIPH Decomposition and Radical Release under Laser Irradiation

Organic dyes, such as indigo carmine, can be degraded by free radicals, and we used indigo carmine to detect whether the photothermal effect of MB NSs could induce free radical generation from AIPH. In brief, water, MB NSs aqueous dispersion (40 μg/mL), AIPH aqueous solution (60 μg/mL), and a mixture of MB NSs and AIPH were combined with indigo carmine solution (50 μg/mL), irradiated with an 808 nm laser (1.0 W/cm^2^) for 5 min. Finally, the absorbance changes at 610 nm for each sample were detected using a spectrophotometer. To reduce the impact of errors and improve the reliability of results, three independent replicates were generally made for in vitro experiments.

### 4.8. Cytotoxicity of MB NSs with AIPH

The human normal liver (L-O2) cells were purchased from KeyGen BioTech, and were cultured in Dulbecco’s modified Eagle’s medium (DMEM) with 10% fetal bovine serum (FBS, Gibco) in an atmosphere with 5% CO_2_ at 37 °C. After growing in 96-well plates for 1 d (10^4^ cell/well), the L-O2 cells were incubated with MB NSs (40 μg/mL) added with different concentrations of AIPH for 24 h, and detect the cell viability of L-O2 cells by using cytotoxicity test kit (MTT assay, KeyGen BioTech, Nanjing, China). To reduce the impact of errors and improve the reliability of results, three independent replicates were generally made for in vitro experiments.

### 4.9. Treatment of Planktonic MRSA In Vitro

Methicillin-resistant *Staphylococcus aureus* (MRSA, ATCC 43300) was grown on Luria-Bertani (LB) agar plate at 37 °C for 12 h. The culture was centrifugated at 12,000 rpm for 3 min. The culture medium was removed and washed with saline 3 times. The bacteria were then dispersed in saline. The absorbance at 600 nm (OD600) of MRSA dispersion was measured for quantifying the number of bacteria (OD600 = 0.1 indicates the concentration of bacteria is 10^7^ CFU/mL). For detecting the antibacterial efficiency of different agents, MB NSs (40 μg/mL) and MB NSs with AIPH (MB NSs: 40 μg/mL; AIPH: 60 μg/mL) were mixed with MRSA dispersion (10^7^ CFU/mL), irradiated with 808 nm laser (1.0 W/cm^2^, 5 min) for the laser-treated group, and incubated for 6 h. After that, the standard plate counting method was used for quantifying the number of alive MRSA after various treatments. To reduce the impact of errors and improve the reliability of results, three independent replicates were generally made for in vitro experiments.

### 4.10. Treatment of Planktonic S. mutans In Vitro

*Streptococcus mutans* (*S. mutans*, ATCC 25923) was grown on brain heart infusion broth (BHI) at 220 rpm with 37 °C for 24 h. The culture was centrifugated at 12,000 rpm for 3 min to remove the culture medium and washed with saline 3 times. Washed bacteria were then dispersed in saline. The absorbance at 600 nm (OD600) of *S. mutans* dispersion was measured for quantifying the number of bacteria (OD600 = 0.1 indicates the concentration of bacteria is 10^9^ CFU/mL). For detecting the antibacterial efficiency of different agents, MB NSs (40 μg/mL) and MB NSs with AIPH (MB NSs: 40 μg/mL, AIPH: 60 μg/mL) were mixed with *S. mutans* dispersion (10^7^ CFU/mL), irradiated with 808 nm laser (1.0 W/cm^2^, 5 min) for the laser-treated group, and incubated for 6 h. After that, the standard plate counting method was used for quantifying the number of alive *S. mutans* after various treatments. To reduce the impact of errors and improve the reliability of results, three independent replicates were generally made for in vitro experiments.

### 4.11. Treatment of S. mutans Biofilm In Vitro

To grow the *S. mutans* biofilm, the bacteria were dispersed in BHI (containing 1% sucrose) and cultured in a 96-well plate for 48 h. After that, the biofilm was treated with MB NSs (40 μg/mL) and MB NSs combined with AIPH (MB NSs: 40 μg/mL, AIPH: 60 μg/mL). The laser-treated group was exposed to an 808 nm laser (1.0 W/cm^2^, 5 min), followed by a 6 h incubation. Subsequently, the treated biofilms were collected and dispersed using ultrasound, and the number of live bacteria was quantified with the standard plate counting method. For SEM analysis, the post-treatment biofilm samples were fixed in a 2.5% glutaraldehyde solution for 30 min, dehydrated using a series of ethanol solutions (15%, 30%, 50%, 75%, and 100%) for 15 min, and imaged with a scanning electron microscope. For 3D confocal laser scanning microscopy (CLSM) analysis, the treated biofilm samples were stained using calcein acetoxymethyl ester (Calcein-AM) solution for 20 min and subsequently imaged with an IX81 laser confocal scanning microscope (Olympus, Allentown, PA, USA). To reduce the impact of errors and improve the reliability of the results, three independent replicates were generally made for in vitro experiments.

### 4.12. Tooth Whitening by Using MBA-CB Gel with Laser Irradiation

All the experiments were approved by the Ethical Committee of Nanjing Stomatological Hospital, Medical School of Nanjing University. Healthy adult third molars were rinsed with PBS to remove the tissue attachments. Subsequently, the teeth were immersed in an indigo carmine solution (10 mg/mL) for one week and then washed with PBS to obtain indigo carmine-stained teeth. These stained teeth were treated with AIPH-CB Gel (AIPH: 2.5 mg/mL), MB-CB Gel (MB NSs: 0.1 mg/mL), and MBA-CB Gel (MB NSs: 0.1 mg/mL, AIPH: 2.5 mg/mL) before being irradiated with an 808 nm laser (1 W/cm^2^) for 60 min. Photographs of the teeth were taken out every 10 min. The color change of indigo carmine-stained teeth after various treatments were calculated by using the Commission International De L’Eclairage (CIELab) system according to the following formula:

$$\Delta E=\sqrt{{\Delta L}^{2}+{\Delta a}^{2}+{\Delta b}^{2}}$$

where Luminance *L* is the difference between light (*L* = 100) and dark (*L* = 0); *a* and *b* are the color values on the red-green axis and blue-yellow axis, respectively; Δ*E* is the color difference. To reduce the impact of errors and improve the reliability of results, three independent replicates were generally made for in vitro experiments.

## Data Availability

The raw data will be available from the corresponding authors upon reasonable request.

## Supplementary Materials

The following supporting information can be downloaded at: https://www.mdpi.com/article/10.3390/gels9070554/s1, Figure S1. The working curve of AIPH aqueous solutions. (a) UV-Vis-NIR spectra of AIPH solutions at different concentrations. (b) The corresponding working curves; Figure S2. Degradation effect of alkyl radicals released from AIPH decomposition on dyes. UV-Vis-NIR spectra and photographs (inset) of methylene blue (a) and rhodamine B (b) under various incubation conditions; Figure S3. Detection of the formation of alkyl radicals by using ABTS. Ultraviolet-visible near-infrared (UV-Vis-NIR) spectra (a,c,e,g) and the corresponding photographs (b,d,f,h) of ABTS aqueous solutions after incubation under different conditions; Figure S4. Laser-activated radical release for indigo carmine degradation. NIR light-triggered degradation of indigo carmine (50 μg/mL) by MB NSs with AIPH (MB NSs 40 μg/mL, AIPH 60 μg/mL). Laser irradiation conditions were kept constant (808 nm, 1.0 W/cm^2^, 5 min) across all experimental groups; Figure S5. Cytotoxicity Assay. Cell viability of L-O2 cells after 24 h incubation with AIPH and MB NSs aqueous solution (AIPH: 0, 60, 120, 240 µg/mL; MB NSs: 40 µg/mL); Figure S6. Three-dimensional (3D) CLSM images of *S. mutans* biofilms stained by Calcein-AM after various treatments (image size: 630 μm × 630 μm); Figure S7. Flowchart of tooth whitening by different Gels; Figure S8. Laser-activated teeth cleaning. The temperature evolution curves of teeth treated with different gels (AIPH-CB Gel, MB-CB Gel, MBA-CB Gel). Laser irradiation conditions were kept constant (808 nm, 1.0 W/cm^2^, 10 min) across all experimental groups.
