# Supplementary material for: On-Demand Free Radical Release by Laser Irradiation for Photothermal-Thermodynamic Biofilm Inactivation and Tooth Whitening
Source: Gels. 2023 Jul 7;9(7):554. doi: 10.3390/gels9070554 (PMC10379348; doi:10.3390/gels9070554)
Supplement: Supplementary file 1 [file gels-09-00554-s001.zip › gels-2457045-supplementary.pdf]

Article

On-Demand Free Radical Release by Laser Irradiation for Photothermal–Thermodynamic Biofilm Inactivation and Tooth Whitening

Qi Zhang [1], Yuan Liu [1], Meng Ding [2], Lihui Yuwen [1,*] and Lianhui Wang [1,*]

[1] State Key Laboratory of Organic Electronics and Information Displays & Jiangsu Key Laboratory for Biosensors, Institute of Advanced Materials (IAM), Nanjing University of Posts and Telecommunications, Nanjing 210023, China; zhangqi_nj@163.com (Q.Z.); lyy657200@163.com (Y.L.)

[2] Nanjing Stomatological Hospital, Medicine School of Nanjing University, Nanjing 210008, China; xzdingmeng@163.com

* Correspondence: iamlhyuwen@njupt.edu.cn (L.Y.); iamlhwang@njupt.edu.cn (L.W.)

Supporting Figures

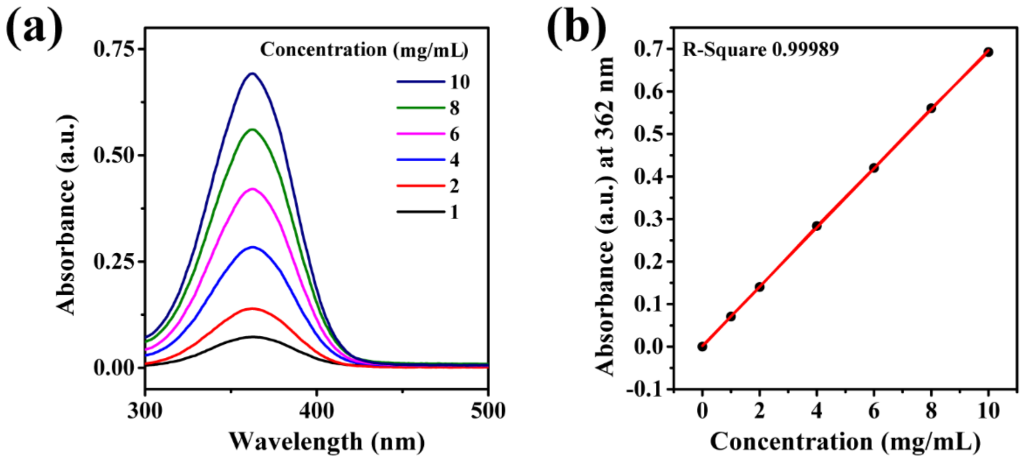

Figure S1. The working curve of AIPH aqueous solutions. (**a**) UV-Vis-NIR spectra of AIPH solutions at different concentrations. (**b**) The corresponding working curves.

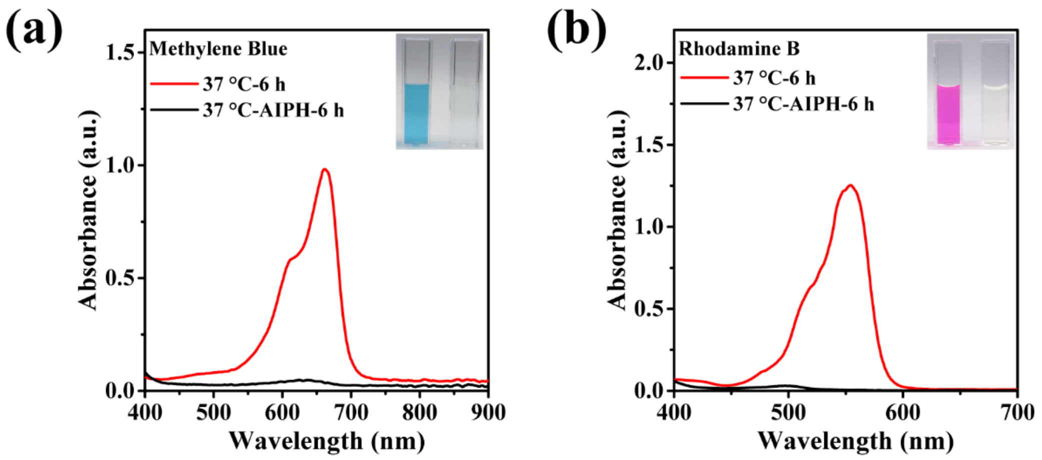

Figure S2. Degradation effect of alkyl radicals released from AIPH decomposition on dyes. UV-Vis-NIR spectra and photographs (inset) of methylene blue (**a**) and rhodamine B (**b**) under various incubation conditions.

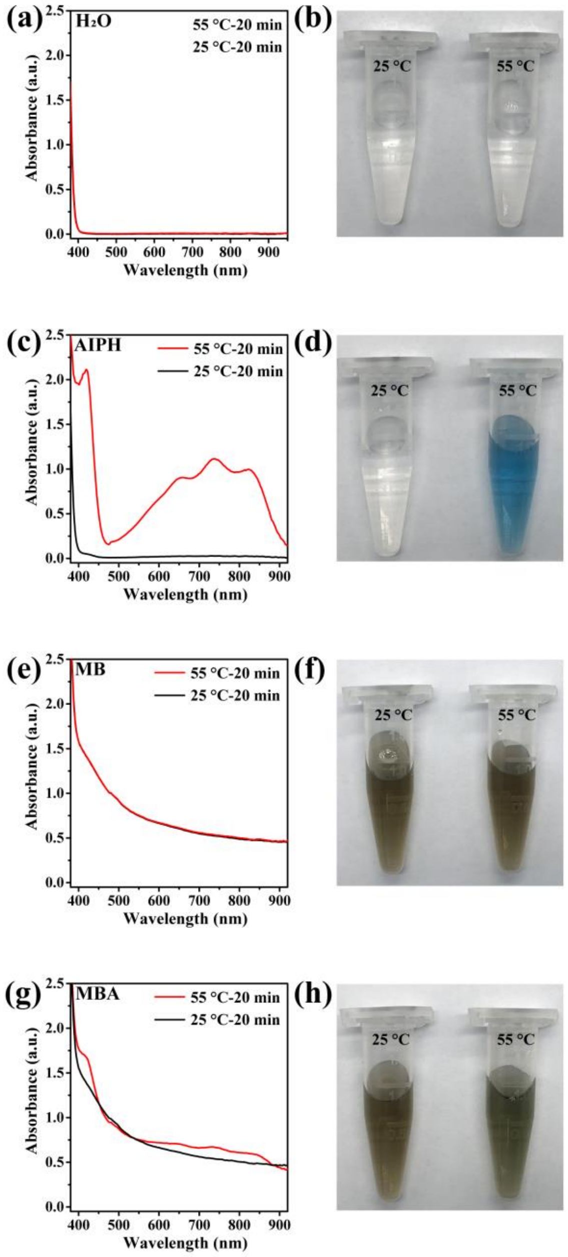

Figure S3. Detection of the formation of alkyl radicals by using ABTS. Ultraviolet-visible near-infrared (UV-Vis-NIR) spectra (**a, c, e, g**) and the corresponding photographs (**b, d, f, h**) of ABTS aqueous solutions after incubation under different conditions.

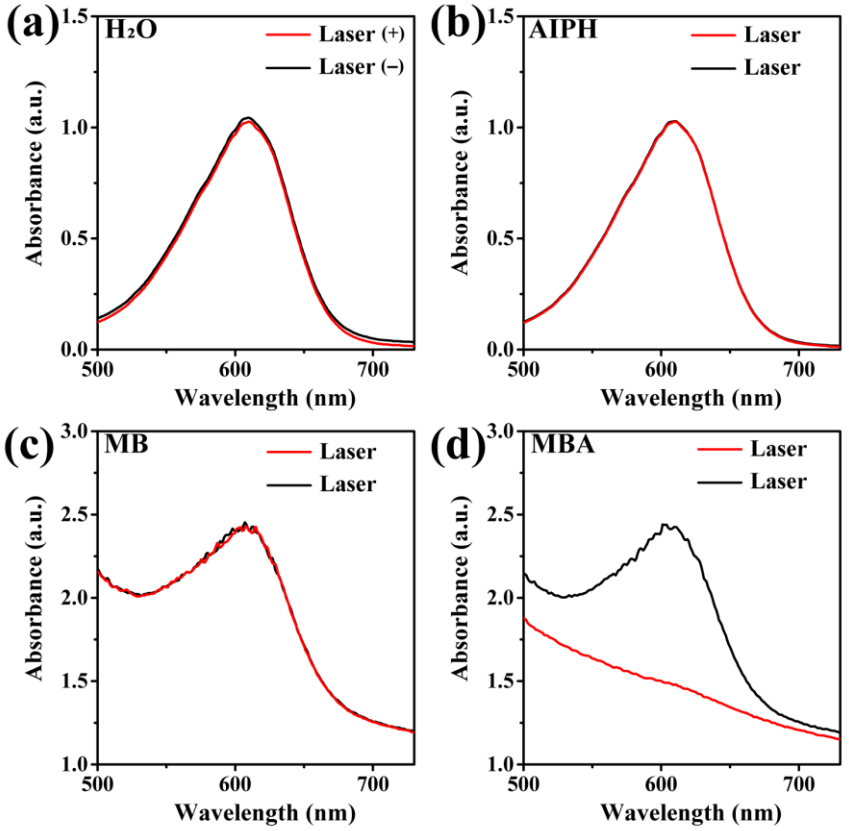

Figure S4. Laser-activated radical release for indigo carmine degradation. NIR light-triggered degradation of indigo carmine (50 μg/mL) by MB NSs with AIPH (MB NSs 40 μg/mL, AIPH 60 μg/mL). Laser irradiation conditions were kept constant (808 nm, 1.0 W/cm^2, 5 min) across all experimental groups.

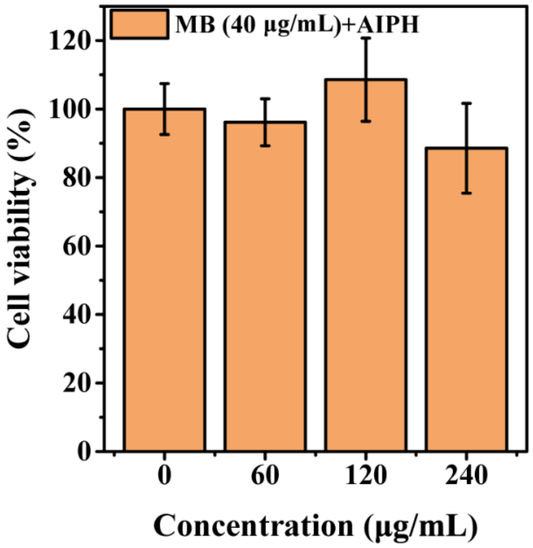

Figure S5. Cytotoxicity Assay. Cell viability of L-O2 cells after 24 hours incubation with AIPH and MB NSs aqueous solution (AIPH: 0, 60, 120, 240 µg/mL; MB NSs: 40 µg/mL).

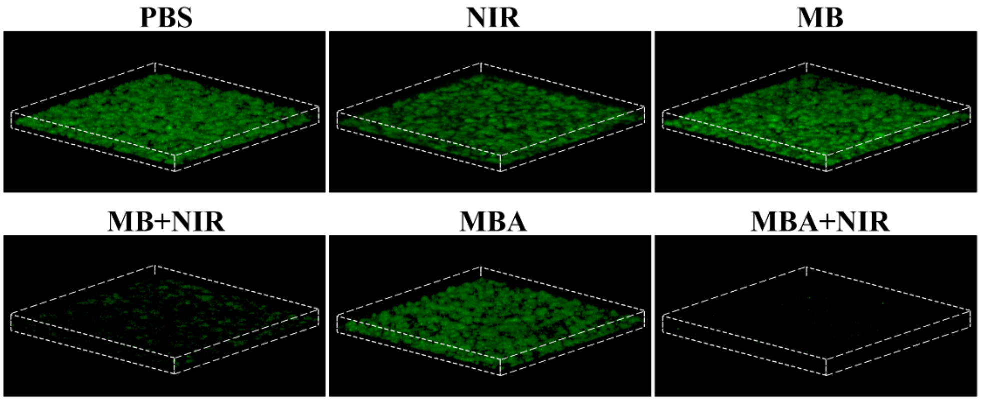

Figure S6. Three-dimensional (3D) CLSM images of *S. mutans* biofilms stained by Calcein-AM after various treatments (image size: 630 μm × 630 μm).

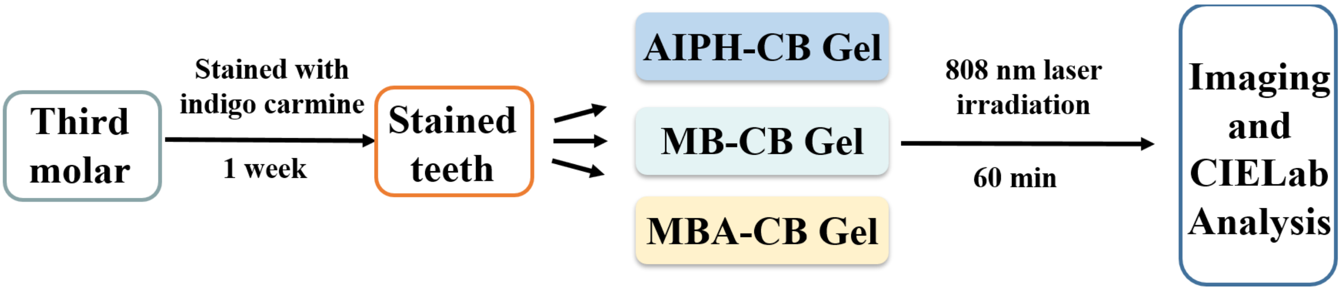

Figure S7. Flowchart of tooth whitening by different Gels.

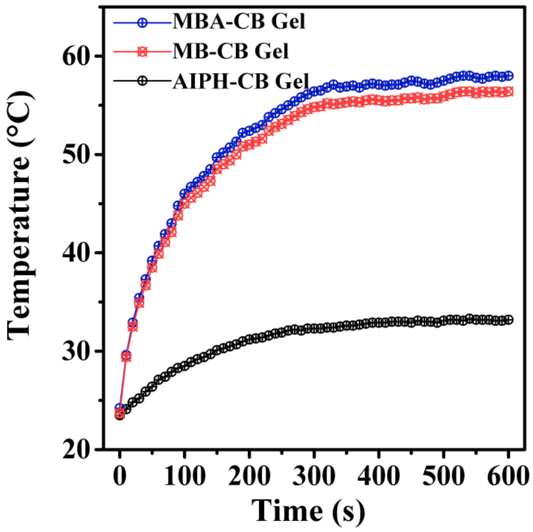

Figure S8. Laser-activated teeth cleaning. The temperature evolution curves of teeth treated with different gels (AIPH-CB Gel, MB-CB Gel, MBA-CB Gel). Laser irradiation conditions were kept constant (808 nm, 1.0 W/cm^2, 10 min) across all experimental groups.
